# Nonverbal pre-performance expressions of professional darts players distinguish between good and poor performance

**DOI:** 10.1038/s41598-021-99729-4

**Published:** 2021-10-11

**Authors:** Philip Furley, Florian Klingner, Daniel Memmert

**Affiliations:** Institute of Training and Computer Science in Sport, German Sport University Cologne, Am Sportpark Müngersdorf 6, 50933 Cologne, Germany

**Keywords:** Psychology, Human behaviour

## Abstract

The present research attempted to extend prior research that showed that thin-slices of pre-performance nonverbal behavior (NVB) of professional darts players gives valid information to observers about subsequent performance tendencies. Specifically, we investigated what kind of nonverbal cues were associated with success and informed thin-slice ratings. Participants (*N* = 61) were first asked to estimate the performance of a random sample of videos showing the preparatory NVB of professional darts players (*N* = 47) either performing well (470 clips) or poorly (470 clips). Preparatory NVB was assessed via preparation times and Active Appearance Modeling using Noldus FaceReader. Results showed that observers could distinguish between good and poor performance based on thin-slices of preparatory NVB (*p* = 0.001, *d* = 0.87). Further analyses showed that facial expressions prior to poor performance showed more arousal (*p* = 0.011, *ƞ*^*2*^_*p*_ = 0.10), sadness (*p* = 0.040, *ƞ*^*2*^_*p*_ = 0.04), and anxiety (*p* = 0.009, *ƞ*^*2*^_*p*_ = 0.09) and preparation times were shorter (*p* = 0.001, *ƞ*^*2*^_*p*_ = 0.36) prior to poor performance than good performance. Lens model analyses showed preparation times (*p* = 0.001, *rho* = 0.18), neutral (*p* = 0.001, *rho* = 0.13), sad (*rho* = 0.12), and facial expressions of arousal (*p* = 0.001, *rho* = 0.11) to be correlated with observers’ performance ratings. Hence, preparation times and facial cues associated with a player’s level of arousal, neutrality, and sadness seem to be valid nonverbal cues that observers utilize to infer information about subsequent perceptual-motor performance.

## Introduction

People are constantly trying to predict how other people are likely to behave. An important source of information in this respect is the nonverbal behavior (NVB) of another person. The present research attempted to extend prior research showing that thin-slices of pre-performance NVB of professional darts players gives valid information to observers about subsequent performance tendencies by investigating what kind of nonverbal cues were associated with success and informed thin-slice ratings. To address this question, participants were first asked to estimate the performance of a random sample of videos showing the preparatory NVB of darts players either performing well or poorly. Next, we sought to explore which objective cues people use to infer performance tendencies by analyzing the video sequences with automated facial coding software.

The terms nonverbal communication (NVC) and NVB are often used interchangeably^1^. Corresponding terms pertinent to the present research are signals versus signs or cues that are given versus cues that are given off^2^. The term signal typically is used to refer to behaviors that belong to a consensually understood code that are shown intentionally (e.g., waving one’s hand when saying goodbye), whereas signs or cues refer to behavior that might be informative but unintended. Although this distinction has proven helpful in research on NVC and NVB [e.g. Hall et al.. for a recent review^1^], it is also problematic due to the difficulty of establishing intentionality and the uncertainty if sender and receiver of nonverbal information share a common understanding of this information. However, as the goal of the present research was to determine differences in NVB as a function of good (hitting a given attempted target) and poor performance (missing the attempted target) of professional darts players immediately prior to throwing darts it seems likely that any potential differences in NVB would pertain to unintentional nonverbal cues.

People are typically categorized in senders and receivers in NVB research. Senders are assumed to encode vast amounts of information within various channels of communication (e.g., visual, auditory, olfactory) which is decoded by receivers (i.e., picking up nonverbal signals and cues, making sense of these, and acting upon them)^3^. The nonverbal cues senders send out likely emerge because of genetic, biological, developmental, and learning factors. These sender cues may be spontaneous or deliberate (posed), and informative to the receivers, albeit this does not have to be the case as plenty of cues are not considered to be informative^1^. Decades of research on nonverbal communication have shown that it is impossible for people to refrain from communicating nonverbally because every cue (or absence of cues) is likely to be interpreted by others^4^. For example, a perceiver might infer that an athlete is focused and concentrated if he has wide open dilated eyes^3^. This inference might be accurate or inaccurate. Importantly, the informational value of any cue or set of cues is probabilistic at best and depends on various other encoded cues, characteristics of the sender and the perceiver, and contextual variables like the nature of the situation (e.g., friendly or hostile)^1^.

The potential informational value of various sender cues can be considered an important line of basic research within the domain of NVC as it enables insights into how senders nonconsciously encode information about themselves and receivers use these cues to draw inferences. A model that has frequently been used to study this process is Brunswick’s^5^ lens model which allows insights into how a criterion variable (e.g., good performance in sports) is correlated with certain nonverbal cues and how these cues are used to inform inferences drawn by a perceiver. The lens model has been used in a variety of domains^6^ and has become a useful framework to explain and investigate how people judge or estimate unobservable characteristics of entities^7–9^. The name lens stems from the assumption that perceivers use a metaphorical lens for indirect perception and inference by focusing on a subset of observable cues in a given situation.

## The present research

Empirical research using the thin slices paradigm has demonstrated that people can make a broad range of accurate inferences from watching short recordings of other people^10–12^. Recent research has transferred the thin slices paradigm to the context of sports given it is widely televised and documented and various outcomes are objectively defined by scores or other performance-related statistics^13^. This research has for example shown that participants can infer the current score based on short recordings (or pictures) of athletes’ NVB during a league or championship game^14,15^ and predict performance tendencies of professional darts players based on thin-slices of pre-performance NVB^16^. The present research attempts to build on the latter study in professional darts as this study did not investigate which cues perceivers used in the studies to inform their inferences of performance tendencies. In addition, the present research addressed a shortcoming of previous research by contrasting NVB as a function of good and poor performance within individual darts players which was not the case in the Furley and Memmert^16^ study.

When conducting lens model studies, it is necessary to decide on what type of behaviors to code and include in the model from the vast amount of options. To this end it is helpful to only include cues that have a known a priori likelihood of being related to the criterion of interest^1^—in this case performance in darts. Hence, a good starting point is if extensive prior research allows one to pick diagnostic cues, that are known to be correlated with the criterion variable^17^. Unfortunately, this is complicated in the current case as there is limited prior research investigating into nonverbal cues that are associated with good and poor performance in darts [^18,19^ for reviews]. However, it is also possible to conduct lens model studies in a more exploratory manner to identify cues^20^ that are associated with a criterion variable and inform observer inferences.

Pertinent to the present research, several lines of investigation within the field of sport psychology point to certain NVBs that might be associated with subsequent perceptual motor performance. First, meta-analyses indicated a connection between emotions and subsequent performance. Specifically, there seems to be evidence that high levels of anxiety and arousal can be negatively associated with athletic performance^21,22^. As emotions often show in a person’s face and previous research has shown that facial information of darts players was sufficient to infer accurate score tendencies^16^, we attempted to test if differential perceptual-motor performance was preceded by distinguishable facial expressions, for example reflecting anxiety or arousal amongst other emotional expressions. Therefore, we analyzed facial muscle activity with the automated facial coding software FaceReader 7 (Noldus Information Technology, Wageningen, The Netherlands).

Another line of investigation within sports has provided initial evidence that the preparatory NVB of athletes might be associated with subsequent performance. Specifically, research has shown that pre-performance preparation times are associated with performance in sports aiming tasks^23^. That is, shorter preparation times have been argued to be indicative of “hastening and hiding” NVB^24^ and therefore can be negatively correlated with subsequent perceptual-motor performance. Hence, it seems feasible that the preparation time of darts players before throwing a dart might be a nonverbal cue that is associated with subsequent performance. Therefore, we decided to analyze whether preparation times might also be a nonverbal cue that is associated with performance tendencies in professional darts and therefore inform observer inferences.

Within lens model research it has been suggested that it is helpful to distinguish between coding molar constructs and/or micro behaviors^1^. Molar constructs require higher levels of abstraction and are typically more holistic in nature (e.g., confidence, insecurity, hectic). Micro behaviors are usually described as not requiring much if any coder inference as they represent more concrete or exact behaviors that are coded descriptively (e.g., an eye blink, certain facial muscle activity). To narrow the scope of exploration in the present research we decided to focally investigate micro behaviors in the face of darts players as FaceReader automatically detects the activation intensity of 20 distinct muscle groups in the face (i.e., ‘action units’), which are used to generate facial expression^25^. In addition, more molar constructs are computed by classifying whole-face expressions into seven basic emotions, following Ekman and Friesen’s Facial Action Coding System (for validity of this classification procedure, see^26^). Further, a combination of action unit intensity and intensity of positive vs. negative emotion was used to infer general levels of valence and arousal (i.e., based on Russell’s circumplex model of affect^27^). Finally, we included the molar construct of preparation time in our model as previous research in sports has indicated that preperformance preparation times are associated with performance in sports aiming tasks^23,24^.

The present research was set up to answer the question of what pre-performance nonverbal cues distinguish between good and poor performance in professional darts and inform observers about performance tendencies of the target players. This research can be considered important in consideration of increased calls of developing a better understanding of naturally occurring nonverbal behavior in high stakes situations^1,3,9,10^, particularly in the domain of human performance^13,18,19^. In line with previous research^16^ we hypothesized that observers’ performance ratings would be higher when viewing the pre-performance NVB of a good performance as opposed to a poor performance of the same player. In addition, we explored differences in facial muscle activity, facial expressions, and preparation time as a function of good vs. poor performance. Finally, we explored if any of these nonverbal cues were significantly correlated to observers’ performance ratings. We did not formulate specific hypotheses for the later exploratory analyses due to a lack of prior research in this domain.

## Method

### Participants

An A prior power analysis revealed that 54 participants (observers for the first part of the study) were necessary to reach sufficient power (0.95) for a small-to-medium effect size (*d*z = 0.5) in a dependent t-test within-subject design and to create a comparable study to previous work in this field^16^. Therefore, 61 participants (32 males and 29 females; *M*age = 33.85; *SD* = 1.489) were recruited and participated in this study (we recruited 5 more participants than the sufficient sample size calculated in case of technical difficulties during data collection). All participants gave informed consent via the experimental software. The study was carried out in accordance with the Helsinki Declaration of 1975 and was approved by the local ethics board at the German Sport University Cologne.

Considering the analyzed darts players, the entire population of the 2017 Professional Darts Corporation (PDC) World darts Championship was used for which sufficient stimulus material was available to meet the inclusion criteria of the study.

### Procedure

#### Stimuli

The used video stimuli in this study were televised video recordings of the first two rounds of the 2017 PDC World darts Championship (for more details on the rules see: www.thedra.co.uk/wp-content/uploads/2015/01/DRA-Rules-final-140115.pdf). These included 29 matches of round one, whereby video footage of three matches of this round were missing, and all matches of round two (16). All matches were screened for the following two performance categories for each player. The high-performance category included all scores which contained two big triples (17–20 points) darts and at least a single (1–20 and 25) or better. Therefore, this category had a possible scoring range from 103 to 180 points. The low-performance category only included single scores or no scores and consisted of a scoring range from 0 to 75. Intentionally thrown bust-throws were excluded, even though these would have met the criterion scoring-wise. A further requirement was to generate at least 10 throws in both performance categories over the course of these first two rounds. This procedure made 50 out of 61 possible players eligible for the further course. Subsequently we numbered all eligible throws of these 50 players in the two performance categories and randomly selected ten high performance and ten low performance throws (1000 throws in total selected) using the webpage www.random.org. The selected throws were then cut and edited with the DaVinci Resolve 16 software.

The created video clips showed the preparatory NVB of one throw, that is three consecutively thrown darts. At the beginning of a throw, darts players always hold three darts in their non-throwing hand (left hand if the player is right-handed) that is hanging loosely beside their body. To initiate the throw of a dart they hand the to-be-thrown dart from their non-throwing hand to their throwing hand in front of their body and raise their throwing arm with the to-be-throw darts in front of their head so that the dart is aligned with the eye of the player and the to-be-hit target^16^.The starting point of every video stimulus was determined as the first frame in which the player made a clear upwards movement with the to-be-thrown dart in his throwing hand. A throwing sequence ending point was determined as the last frame before the dart left the hand. All three pre-performance throwing sequences of a throw were merged together to one video clip. A video clip was not considered eligible for the study, if the footage of a selected throw did not show the player and his pre-performance NVB of all three individual thrown darts within a throw. In that case the video clip was excluded and replaced by the subsequent number on the list of all eligible throws created by the webpage www.random.org. The broadcasted scoreboard and the dartboard, if it was shown in the split-screen, were blacked out and the sound was muted. These two possible sources of information were excluded to ensure that participants would solely make their judgements based on the preparatory NVB and not for example based on crowd noise. This procedure created comparable stimuli to previous research on preparatory NVB in darts^16^.

In the next step, all clips were pre-analysed with the Noldus FaceReader 7.1 software, which was necessary for the data analyses. If a clip could not be analysed due to insufficient facial information in the video, that clip would be excluded from the data set and again was replaced by the next listed one. If these exclusions led to a player having less than 10 video clips in one of the two performance categories, this player was removed from the data set. As a result of this process the final data set consisted of 47 players with 10 valid video clips per performance category (940 video clips in total). All clips consisted of a resolution of 480 × 720 pixel with a frame rate of 30,00 fps (frames per second). The average duration of a video clip was 4.436 s (*SD* = 1.351).

### Measures

Using Active Appearance Modelling^28^, FaceReader calculates the activation intensity of 20 distinct muscle groups in the face (i.e., ‘action units’), which are used to generate facial expression^25^. The validity of coding action unit intensities has been reported as acceptable for most action units^29^.

#### Action unit (AU) intensities

FaceReader automatically computes continues variables reflecting the intensity of the following AUs (for description and illustration of individual AUs see: https://www.noldus.com/applications/facial-action-coding-system: AU 1. Inner Brow Raiser (Muscular basis: frontalis, pars medialis); AU 2. Outer Brow Raiser (Frontalis, pars lateralis); AU 4. Brow Lowerer (depressor glabellae, depressor supercilii, and corrugator supercilia); AU 5. Upper Lid Raiser (levator palpebrae superioris, and superior tarsal muscle); AU 6. Cheek Raiser (Orbicularis oculi, pars orbitalis); AU 7. Lid Tightener (Orbicularis oculi, pars palpebralis); AU 9. Nose Wrinkler (Levator labii superioris alaeque nasi); AU 10. Upper Lip Raiser (Levator labii superioris, caput infraorbitalis); AU 12. Lip Corner Puller (zygomaticus major); AU 14. Dimpler (Buccinator is the underlying muscle); AU 15. Lip Corner Depressor (Depressor anguli oris); AU 17. Chin Raiser (mentalis); AU 18. Lip Pucker (incisivii labii superioris and incisivii labii inferioris); AU 20. Lip Stretcher (risorius w/platysma); AU 23. Lip Tightener (orbicularis oris); AU 24. Lip Pressor (orbicularis oris); AU 25. Lips Part (depressor labii inferioris, or relaxation of mentalis or orbicularis oris); AU 26. Jaw drop (masseter; relaxed temporalis and internal pterygoid); AU 27. Mouth Stretch (pterygoids and digastric); AU 43. Eyes Closed (relaxation of Levator palpebrae superioris).

Action Units were exported as continuous variables with FaceReader ranging from 0 to 1. Values from 0.00 to 0.16 are classified as Trace, 0.16–0.26 as Slight, 0.26–0.58 as Pronounced, 0.58–0.90 as Severe, and 0.90–1 as Max Intensity for every video frame^25,28^. The dependent variable was the mean of every analyzed frame in the respective video^29^.

#### Emotional facial expressions

Subsequently, whole-face expressions were classified in seven basic emotions (Happiness, Sadness, Anger, Surprise, Fear, Disgust, and Neutral) by the FaceReader algorithm, following Ekman and Friesen’s Facial Action Coding System (for validity of this classification procedure, see^26,29^). We used the default settings using a general face model with continuous calibration. The dependent variable was the mean of the respective basic emotion of every analyzed frame in the respective video.

#### Valence and arousal

In addition, a combination of action unit intensity and intensity of positive vs. negative emotion is used to infer general levels of valence and arousal (i.e., based on Russell’s circumplex model of affect^27^). The dependent variable was the mean of the valence and arousal scores in every analyzed frame in the respective video.

#### Preparation time

Finally, we computed the cumulative preparation time of the three individual darts that make up a throw by counting the frames of the respective video clips. The starting point of every individual dart was determined when a player made a clear upwards movement with one dart in his throwing hand to initiate the beginning of the throwing motion and ended with the last frame before the dart left the hand. The frames for the individual darts were summed up for the three darts of a throw as the final measure of preparation time.

#### Software and experimental procedure

The experiment was programmed as a desktop version with the PsychoPy2 software^30^. For each trial the software randomly sampled 14 players from the pool of 47 players and respectively seven video clips from each performance category. Hence, every observer participant viewed and rated a total of 196 selected videos in random order. This approach ensured that the results of this experiment did not depend on a specific combination of the stimuli. The software ensured that video clips were only shown once per participant. The participants were asked after each shown video clip to estimate the score of the throw (all three darts together as this is how it is scored in competition) based on the pre-performance NVB of the dart player. Therefore, a digital slider bar was presented with a 180 points scale, where the left pole represented 0 points (the poorest possible performance) and the right pole represented 180 points (highest possible performance). The starting point was always in the middle of the bar (90 points) and the participants had to move the mouse curser along the bar for their rating to log in the estimated score by clicking the left mouse button. The exact number of the estimated score was not presented to the participants during this process. We used this procedure as the scores correspond to the potential scoring range in professional darts and this slider was used in previous research^16^ that the present study aimed to extend.

At the beginning of the experiment the participants completed a questionnaire that collected demographical data and gave their consent. Every participant was tested individually on a 15-in. screen laptop. After the introduction and one practice trial the experimenter left the testing room to reduce distraction for the participant. At the end of the completed experiment the participants were informed about the purpose of the study. The whole experiment lasted about 25 min.

#### Statistical analyses

All data were checked for normality with Kolmogorov–Smirnov-tests. We first ran a dependent t test to compare performance ratings as a function of whether the darts players scored high or low. To address the question of which nonverbal cues differed as a function of performance, we first ran a series Wilcoxon signed-ranks tests on the averaged FaceReader variables and preparation times in the high and low performance categories. We used these non-parametric tests as most of the FaceReader variables differed significantly from normality. To address the question of which nonverbal cues were correlated with observer performance ratings and darts’ players performance, we ran a series of bivariate Spearman rho correlations between the FaceReader measures, darts players performance (the actual score achieved in the videos), and preparation times in all 940 videos used in the present research (47 darts players × 2 performance categories × 10 videos). Finally, we ran two multiple regression analyses using the forward method, once using the molar independent variables and once the micro facial behaviors as predictors of the ‘mean observer performance ratings’ to explore cue utilization of the participants. All statistical analyses were computed with IBM SPSS Statistics 27.

### Disclosure

The authors declare that (1) (a) the total number of excluded observations and (b) the reasons for making these exclusions have been reported in this manuscript; (2) that all independent variables or manipulations, whether successful or failed, have been reported in the manuscript; (3) that all dependent variables or measures that were analyzed for this article’s target research question have been reported in in the manuscript/online supplements; (4) that (a) how sample size was determined and (b) our data-collection stopping rule have been reported in this manuscript.

## Results

**Figure 1:**
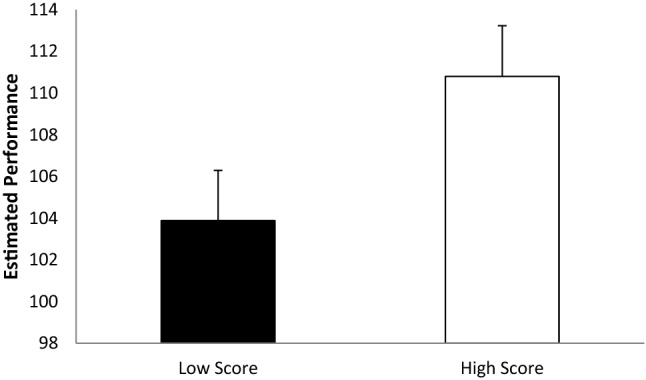
Participants mean score ratings as a function of actual performance (error bars represent standard errors).

The descriptive statistics are summarized in Fig. 1.

A dependent t test revealed a significant difference in participant estimates as a function of whether the darts players scored high or low (*t*(61) = 7.791, *p* = 0.001, *d* = 0.870). This effect was also evident when comparing the average participant ratings in the high and low score category for the 47 darts players and subjected these to a dependent t test (*t*(46) = 8.069, *p* = 0.001, *d* = 1.293). Hence, participants predicted a higher score based on the darts’ players NVB if they actually did perform better. The next series of analyses sought to illuminate what nonverbal cues differed as a function of darts players’ performance informed the observers’ performance ratings.

### Cue validity: which nonverbal cues differed as a function of performance?

Table 1 shows the results of Wilcoxon signed-ranks tests on the averaged FaceReader variables and preparation times. None of the micro facial behaviors significantly differed as a function of performance, except for AU 26, Jaw drop (masseter; relaxed temporalis and internal pterygoid). On the aggregated molar constructs computed by FaceReader some significant differences emerged. Specifically, lower performance was associated with a more sad and scared facial expression than was shown on average in the high-performance throws. Further, darts players showed on average less physiological arousal in their facial expressions before successful performances compared to lower performances. However, the most pronounced difference between the high and low performance categories was evident in the preparatory time that dart players took. On average darts players took significantly longer when preparing for high performance throws as compared to low performance throws.

**Table 1:** Descriptive statistics and inferential statistics (Wilcoxon tests) as a function of high and low performance for all dart throws per shown dart player. Statistics were calculated on the mean values of the high and low performance of each players dart throws.

	Descriptive				Inferential		
	High performance		Low performance				
	*M*	*SD*	*M*	*SD*	*Z*	*p*	*ƞ*^2^
**Action unit intensity (micro)**							
Inner brow raiser	0.0191	0.0587	0.0229	0.0787	− 0.574	0.566	0.006
Outer brow raiser	0.0045	0.0276	0.0034	0.0220	− 0.734	0.463	0.030
Brow lowerer	0.0504	0.0911	0.0428	0.0871	− 0.544	0.587	0.018
Upper lid raiser	0.0517	0.1546	0.0608	0.1724	− 0.730	0.465	0.036
Cheek raiser	0.0047	0.0203	0.0028	0.0152	− 0.982	0.326	0.067
Lid tightener	0.0500	0.1092	0.0523	0.1068	− 0.267	0.789	0.002
Nose wrinkler	0.0001	0.0005	0.0011	0.0055	− 1.172	0.241	0.031
Upper lip raiser	0.0143	0.0676	0.0283	0.1085	− 1.743	0.081	0.064
Lip corner puller	0.0260	0.0656	0.0376	0.1156	− 0.048	0.962	0.011
Dimpler	0.0417	0.0834	0.0397	0.0668	− 0.060	0.952	0.001
Lip corner depressor	0.2545	0.2662	0.2634	0.2480	− 1.496	0.135	0.007
Chin raiser	0.0715	0.1116	0.0687	0.1033	− 0.024	0.981	0.002
Lip puckerer	0.0023	0.0088	0.0041	0.0143	− 0.341	0.733	0.010
Lip stretcher	0.0025	0.0062	0.0016	0.0052	− 0.893	0.372	0.015
Lip tightener	0.0086	0.0247	0.0085	0.0258	− 0.608	0.543	0.000
Lip pressor	0.0324	0.1090	0.0417	0.1179	− 0.638	0.524	0.032
Lips part	0.0710	0.1189	0.0835	0.1243	− 1.393	0.164	0.024
Jaw drop	0.0299	0.0579	0.0412	0.0698	**− 2.211**	0.027*	0.**046**
Mouth stretch	0.0039	0.0106	0.0086	0.0387	− 0.094	0.925	0.016
Eyes closed	0.1100	0.1849	0.1134	0.1973	− 0.698	0.485	0.002
**Molar constructs**							
Emotion							
Neutral	0.5581	0.2163	0.5830	0.2396	− 1.407	0.159	0.043
Happy	0.0463	0.0888	0.0504	0.0859	− 0.370	0.711	0.019
Sad	0.1700	0.1328	0.1801	0.1331	**− 2.053**	0.040*	0.042
Angry	0.0945	0.0668	0.0964	0.0704	− 0.519	0.604	0.008
Surprised	0.0278	0.0199	0.0292	0.0217	− 0.783	0.434	0.011
Scared	0.0228	0.0299	0.0255	0.0320	**− 2.614**	0.009**	**0.086**
Disgusted	0.0144	0.0159	0.0150	0.0165	− 1.026	0.305	0.007
Valence	− 0.0017	0.0018	− 0.0018	0.0018	− 1.661	0.097	0.011
Arousal	0.0040	0.0018	0.0043	0.0018	− **2.540**	0.011*	**0.095**
Preparation time	4.582	1.171	4.289	1.028	**− 4.206**	0.001**	**0.363**
Observer performance rating	110.900	8.833	103.925	8.678	**− 5.556**	0.001**	**0.586**

Significant differences are printed in bold and marked with * for *p* < .05 and ** for *p* < .01.

Given these significant differences in the facial expressions of sadness, anxiety, arousal, and preparation time, we next explored correlations between observers’ performance ratings, actual performance, and the darts players nonverbal cues in all 940 experimental video clips.

### Cue utilization: what nonverbal cues were correlated with observer performance ratings and darts’ players performance?

The only nonverbal cue variables that were significantly correlated to both observers’ performance ratings and to darts players performance was pre-performance preparation time, the neutrality of the face, the sadness of the face, and the displayed arousal in the face (see Table 2). However, all the significant correlations were small. There were some significant bivariate correlations between action unit intensities and observer performance ratings: a higher intensities of the upper lid raiser was significantly correlated with higher performance ratings. Also, a lower intensity of the lip corner puller, dimpler, and chin raiser was associated with higher performance ratings. Although this might indicate that differences in these action units might have been used by observers to inform their ratings, the lack of a correlations between these action units and performance of darts players indicates that these cues were not valid cues to help observers in achieving higher accuracy in their performance ratings. Observers’ performance ratings were also correlated with the disgusted variable computed by FaceReader (more disgusted was associated with slightly higher performance ratings). However, the lack of correlation with performance suggests that this was not a valid cue that helped observers in achieving higher accuracy in their performance ratings. On the other hand, scared facial expressions were valid cues but these did not seem to be used by observers to infer score tendencies.

**Table 2:** Statistics (Spearman correlations) were calculated on the values of the mean rating and the points scored as performance measure of all Videos.

	*N*	Correlation			
		Mean rating		Performance	
		*rho*	*p*	*rho*	*p*
**Action unit intensity (micro)**					
Inner brow raiser	940	0.002	0.952	− 0.039	0.226
Outer brow raiser	940	0.004	0.907	− 0.013	0.688
Brow lowerer	940	0.004	0.909	0.005	0.889
Upper lid raiser	940	0.078*	**0.016**	0.005	0.886
Cheek raiser	940	− 0.046	0.163	0.033	0.315
Lid tightener	940	0.060	0.068	0.064	0.051
Nose wrinkler	940	0.037	0.256	0.004	0.906
Upper lip raiser	940	0.020	0.543	0.012	0.703
Lip corner puller	940	− 0.068*	0.**037**	0.001	0.985
Dimpler	940	− 0.077*	0.**018**	0.018	0.579
Lip corner depressor	940	− 0.046	0.161	− 0.012	0.707
Chin raiser	940	− 0.093*	0.**004**	− 0.044	0.176
Lip puckerer	940	− 0.003	0.936	− 0.003	0.932
Lip stretcher	940	0.010	0.752	− 0.001	0.974
Lip tightener	940	− 0.018	0.587	0.042	0.200
Lip pressor	940	− 0.033	0.311	0.038	0.473
Lips part	940	− 0.028	0.400	− 0.023	0.400
Jaw drop	940	− 0.055	0.094	− 0.053	0.107
Mouth stretch	940	0.046	0.160	0.005	0.878
Eyes closed	940	0.013	0.680	0.026	0.426
**Molar constructs**					
Emotion					
Neutral	940	− 0.129**	0.**001**	− 0.075*	0.**021**
Happy	940	− 0.038	0.249	− 0.009	0.786
Sad	940	− 0.117*	0.**001**	− 0.073*	0.**024**
Angry	940	− 0.058	0.075	− 0.058	0.075
Surprised	940	0.010	0.749	0.043	0.188
Scared	940	0.016	0.622	− 0.077*	0.**018**
Disgusted	940	0.067*	0.**040**	0.003	0.915
Valence	940	0.044	0.178	0.064	0.050
Arousal	940	− 0.106**	0.**001**	− 0.089**	0.**006**
Preparation time	940	0.182**	0.**001**	0.175**	0.**001**

Significant correlations are printed in bold and marked with * for *p* < .05 and ** for *p* < .01.

To further illuminate which variables observers used to inform their performance ratings we ran two separate multiple regression analysis on the (1) more molar expressions and preparation time and the (2) micro facial behaviors.

#### Cue utilization: regression analysis of molar constructs

A multiple regression analysis using the forward method with the ten molar independent variables (neutral, happy, sad, angry, surprised, scared, disgusted, valence, arousal, preparation time) on the dependent variable ‘mean observer performance ratings’ showed that only preparation time significantly predicted observer ratings. This model could explain *R*^*2*^ = 0.036 in variance (i.e. 3–4% variance explained) of observer ratings, (*F*(1, 938) = 34.574, *p* = 0.00001). The preparation time was a significant single predictor of observer performance evaluations, *β* = 0.189, *t* = 5.880, *p* = 0.00001, while all other molar emotion variables did not significantly explain variance of the model (neutral: *β* = − 0.019; *t* = 0.552, *p* = 0.581; happy: *β* = − 0.053; *t* = − 1.656, *p* = 0.098; sad: *β* = − 0.017; *t* = − 0.516, *p* = 0.690; angry: *β* = 0.042; *t* = 1.228, *p* = 0.220; surprised: *β* = 0.033; *t* = 1.013, *p* = 0.311; scared: *β* = 0.009; *t* = 0.295, *p* = 0.768; disgusted: *β* = − 0.005; *t* = − 0.155, *p* = 0.877; valence: *β* = − 0.044; *t* = − 1.322, *p* = 0.186; arousal: *β* = − 0.029; *t* = − 0.872, *p* = 0.384).

#### Cue utilization: regression analysis of micro facial behavior

A multiple regression analysis using the forward method with the twenty action units as independent variables (inner brow raiser, outer brow raiser, brow lowerer, upper lid raiser, cheek raiser, lid tightener, nose wrinkle, upper lip raiser, lip corner puller, dimpler, lip corner depressor, chin raiser, lip puckerer, lip stretcher, lip tightener, lip pressor, lips part, jaw drop, mouth stretch, eyes closed) was used to test if facial movements significantly predicted observer ratings of performance. The results of the regression indicated that three predictors explained 2.3% of the variance (*R*^*2*^ = 0.023, *F*(3, 939) = 7.466, *p* = 0.001). It was found that the activity of the upper lid raiser significantly predicted observer performance ratings *β* = 0.112; *t* = 3.478, *p* = 0.001), as did the dimpler *β* = − 0.088; *t* = − 2.687, *p* = 0.007), and the nose wrinkler *β* = 0.068; *t* = 2.067, *p* = 0.039). None of the other action unit intensities significantly contributed to the model (all *p* > 0.081).

## Discussion

The central aim of the study was to extend prior research that showed that thin-slices of pre-performance NVB of professional darts players give valid information to observers about subsequent performance tendencies by investigating what kind of nonverbal cues were associated with success and informed thin-slice ratings. The present findings replicate the findings from Furley and Memmert^16^ while making sure that high and low performances were contrasted within individual darts players which was not the case in Furley and Memmert^16^. Stated differently, observers were able to correctly differentiate high and low performance of individual darts players based on short glimpses of preparatory NVB. In addition, the present research found that pre-performance preparation times, neutral, sad, and facial expressions of arousal were both correlated to subsequent performance and significantly influenced observer ratings. Hence, the most important finding regarding Brunswick’s^5^ lens model that emerged from the present research was that good performance in professional darts is correlated with the preparation times, expressions of neutrality, sadness, and arousal (cue validity) and that observers use these cues to inform inferences about the respective performance (cue utilization). However, it is important to mention that all of these correlations are small and only explain small proportions of variance.

The association between preparation times and performance in darts is supportive of research in soccer penalties that has found that shorter preparation times before shooting high stakes penalties in penalty shootouts during major tournaments is correlated with worse performance^23^. In addition, the finding that this nonverbal cue was used by observers to inform their performance ratings can be regarded as supportive of research that has shown that shorter preparation times by soccer players prior to shooting a penalty kick lead to more negative impressions among observers who view this preparatory NVB^24^. Hence, from an applied perspective it seems advisable for darts players (and potentially athletes in general) to avoid rushing through their pre-performance preparation and taking their time. However, further research is needed in determining the optimal preparational timing in different sport contexts. In addition, a potential limitation of the present research was that preparation times were calculated based on broadcasted footage for television. Therefore, the editing and cutting of the video material could not be controlled and we had to use the broadcasted video material for this study.

Although, there were differences in emotional facial expressions prior to high performance or low performance in the amounts of anxiety, sadness, and arousal that darts players expressed the present analyses did not find anxiety to be correlated with observers’ performance ratings. Nevertheless, this finding is intriguing as it indicates that via Active Appearance Modelling^28^, FaceReader can detect systematic changes in the faces of professional darts players prior to throwing darts that are associated with subsequent performance. Although we acknowledge that this finding needs to be further scrutinized, it is in line with abundant theorizing within sport psychology on the relationship of anxiety and arousal on perceptual-motor performance. While there is no straight-forward relationship between anxiety and performance or arousal and performance^22,31,32^, the present research indicates that certain facial expressions that have been linked to relatively higher levels of anxiety, sadness, and arousal are negatively associated with perceptual-motor performance in darts.

Another preliminary finding that might point to an interesting avenue for future research was that the certain muscles around the eye (i.e., AU 5. Upper Lid Raiser [levator palpebrae superioris, and superior tarsal muscle] and AU 14. Dimpler (Buccinator), 9. Nose Wrinkler (Levator labii superioris alaeque nasi); explained about 2.3 per cent of variance in observers’ performance ratings in a regression analysis. Although these facial action units cannot be regarded as valid cues as they were not correlated to performance, a higher activity in these muscles might be related to better performance evaluations. In this respect, applied research might want to investigate the possibility if athletes can deliberately use certain facial expression to create favorable impressions in observers like opponents or spectators^2,18,19^. We acknowledge that this is speculative at present but seems to be an important avenue for applied research in the field of sports. One reason for the low proportion of variance explained could be that the expressions of darts players before throwing darts is mainly neutral and does not show strong emotional expressions^16^. Therefore, the present research was not likely to explain large portions of variance. Research investigating different nonverbal expressions of darts players as a function of winning or losing a match are arguably more likely to reveal larger effect sizes^18^.

Research on NVB and NVC has shown that different nonverbal cues are often correlated and collectively serve to inform observers about other people which has led to the theoretical concept of nonverbal response system coherence^33^. In this respect, previous thin-slices research in sports has shown that different nonverbal cues (in an athlete’s face, body, and kinematics) informed observers about the current state of the athlete^15^. Hence, we acknowledge that the present research only explored a small range of potential nonverbal cues that were mainly derived from the automated facial action coding software FaceReader. We therefore consider it likely that there are other nonverbal cues that are linked to perceptual-motor performance in darts and in other sports that were not investigated in the present research. As the lens model has been used in a variety of domains^6^ and has become a useful framework to explain and investigate how people judge or estimate unobservable characteristics of entities^7–9^, we think that the present extension of the lens model to the domain of NVB in sports might have heuristic value. In line with renewed calls of measuring in situ social behavior^34^, we consider that the broadly televised and documented sports competitions offer a compelling behavioral window into psychological processes, particularly in the domain of emotions and NVB^13,32^.

In conclusion, humans are constantly displaying NVB—whether they want to or not^35^—that are associated with certain internal states and therefore inform other people how they are currently feeling and likely to behave. In this respect, preparation times and facial expressions of anxiety, sadness, and arousal prior to perceptual-motor performance in professional darts can be considered as a part of nonverbal cues that observers can use to infer performance tendencies in darts.

## Data Availability

All data will be made available at reasonable request to the corresponding author.
